# Supplementary material for: A Likelihood Approach for Real-Time Calibration of Stochastic Compartmental Epidemic Models
Source: PLoS Comput Biol. 2017 Jan 17;13(1):e1005257. doi: 10.1371/journal.pcbi.1005257 (PMC5240920; doi:10.1371/journal.pcbi.1005257)
Supplement: S1 File — (TAR.GZ) [file pcbi.1005257.s014.tar.gz › HSPH_Online-SI-Revision/IPoi09/n100-mild/n100-mild_BENCH_table-4.pdf]

1	R0	{1.24125, 3.38982}	{1.40091, 4.2425}	{{1.40116, 4.24222}, {1.40116, 4.24222}}	{12.8632, 25.1541}	inf	1007.	3537.44	{1152., 4712.}	261.503	0.572464	newly	2409.	5911.33	{2434., 7659.}	155.828	0.437572	Reff	1.16056	1.09104	{0.907792, 1.34523}	13.3732	ar	34 926.	51 165.2	{50 445., 51 984.}	46.496
2	R0	{1.29554, 4.51878}	{1.40116, 4.24222}	{{1.40116, 4.24222}, {1.40116, 4.24222}}	{8.15259, 6.12024}	inf	1718.	3595.15	{1798., 4482.}	119.599	0.380238	newly	2989.	5772.37	{2454., 7312.}	103.581	0.34206	Reff	1.16829	1.01705	{0.839645, 1.30175}	17.1028	ar	42 828.	51 167.2	{50 445., 51 984.}	19.4713
3	R0	{1.2443, 3.64292}	{1.36035, 3.22945}	{{1.36035, 3.22945}, {1.36035, 3.22945}}	{9.32653, 11.35}	inf	1255.	3235.85	{2325., 3789.}	157.837	0.403542	newly	2853.	6903.3	{4533., 8296.}	141.966	0.375241	Reff	1.15569	1.01423	{0.81606, 1.20584}	14.1606	ar	37 087.	47 805.2	{46 157., 48 882.}	28.9
4	R0	{1.38465, 3.43331}	{1.47917, 2.88072}	{{1.43965, 2.60601}, {1.54098, 3.31039}}	{6.82618, 16.095}	inf	1365.	4131.	{1662., 6057.}	206.851	0.460804	newly	3916.	11 346.6	{6055., 14 316.}	193.836	0.463725	Reff	1.30897	1.21143	{1.01595, 1.42299}	11.7411	ar	49 511.	57 071.5	{53 540., 61 309.}	15.2703
5	R0	{1.25409, 4.1068}	{1.4761, 4.12341}	{{1.4761, 4.12341}, {1.4761, 4.12341}}	{17.7029, 0.404451}	inf	1456.	4936.23	{1725., 6007.}	239.027	0.503804	newly	2839.	8485.04	{4171., 10 022.}	198.874	0.460328	Reff	1.15606	1.08486	{0.864257, 1.38873}	12.9092	ar	38 571.	56 955.5	{56 082., 57 806.}	47.6639
6	R0	{1.28821, 4.2016}	{1.42949, 4.01966}	{{1.42949, 4.01966}, {1.42949, 4.01966}}	{10.9672, 4.33026}	inf	1742.	3869.64	{2251., 4857.}	129.104	0.364289	newly	3382.	6695.18	{4376., 8513.}	104.336	0.312969	Reff	1.16363	1.04634	{0.841026, 1.30309}	16.2572	ar	41 468.	53 399.4	{52 765., 53 977.}	28.7725
7	R0	{1.24369, 2.56007}	{1.37363, 2.81014}	{{1.37363, 2.81014}, {1.37363, 2.81014}}	{10.4479, 9.76809}	inf	601.	2598.68	{926., 3975.}	341.318	0.651842	newly	2262.	7264.91	{3191., 9823.}	230.094	0.552153	Reff	1.20376	1.17807	{1.0312, 1.31437}	9.31742	ar	38 603.	48 965.7	{48 188., 49 769.}	26.8444
8	R0	{1.232, 3.53942}	{1.67405, 7.28757}	{{1.67405, 7.28757}, {1.67405, 7.28757}}	{35.8807, 105.897}	inf	1387.	6924.03	{2099., 9526.}	402.195	0.659117	newly	3108.	7134.21	{2698., 9058.}	137.174	0.367296	Reff	1.11866	1.25975	{0.968756, 1.57925}	18.1397	ar	34 555.	67 952.9	{67 276., 68 619.}	96.6513
9	R0	{1.36262, 3.85631}	{1.53501, 4.02511}	{{1.53501, 4.02511}, {1.53501, 4.02511}}	{12.6514, 4.37724}	inf	1592.	4837.77	{1813., 6819.}	210.603	0.477566	newly	3831.	9479.	{4690., 12 053.}	153.525	0.40422	Reff	1.27209	1.23994	{1.08691, 1.44363}	10.2951	ar	48 410.	60 554.2	{60 065., 61 260.}	25.0861
10	R0	{1.28702, 4.67968}	{1.38889, 4.25615}	{{1.38889, 4.25615}, {1.38889, 4.25615}}	{7.91518, 9.05041}	inf	1874.	3051.	{1454., 4248.}	71.7068	0.226317	newly	3114.	4640.	{2003., 7027.}	62.024	0.205808	Reff	1.13924	0.892321	{0.738556, 1.0996}	22.7188	ar	41 092.	50 357.8	{49 757., 51 023.}	22.5489
11	R0	{1.35752, 3.47129}	{1.739, 7.10524}	{{1.739, 7.10524}, {1.739, 7.10524}}	{28.1012, 104.686}	inf	974.	4779.79	{1400., 8517.}	393.998	0.624243	newly	2552.	5724.38	{1939., 9385.}	135.748	0.355323	Reff	1.29461	1.5111	{1.29889, 1.67579}	17.3571	ar	44 669.	70 793.5	{70 290., 71 335.}	58.4847
12	R0	{1.35929, 5.83647}	{1.68297, 7.5059}	{{1.68297, 7.5059}, {1.68297, 7.5059}}	{23.8124, 28.6034}	inf	2459.	7871.71	{3510., 9761.}	220.945	0.480899	newly	3330.	7480.71	{4159., 9061.}	125.915	0.340486	Reff	1.20052	1.13624	{0.860604, 1.52129}	18.3578	ar	46 715.	68 311.3	{67 696., 68 803.}	46.2299
13	R0	{1.28019, 3.29908}	{1.51438, 4.33099}	{{1.50263, 4.22172}, {1.50263, 4.22172}}	{18.2935, 31.2788}	inf	1185.	4578.21	{2777., 6454.}	286.347	0.554003	newly	3104.	8276.04	{5842., 10 780.}	166.625	0.407931	Reff	1.21037	1.22302	{0.980316, 1.43488}	12.3086	ar	40 941.	59 258.9	{57 802., 61 217.}	44.7423
14	R0	{1.36661, 3.48318}	{1.4804, 3.12918}	{{1.4804, 3.12918}, {1.4804, 3.12918}}	{8.32644, 10.1631}	inf	1869.	3782.85	{588., 5895.}	126.71	0.399961	newly	4569.	9398.85	{2400., 13 150.}	124.276	0.381965	Reff	1.26507	1.21043	{0.929114, 1.44962}	12.4749	ar	48 672.	57 005.2	{56 101., 57 732.}	17.1212
15	R0	{1.35865, 3.84123}	{2.60929, 14.519}	{{2.43464, 12.8735}, {2.80057, 16.2348}}	{92.0504, 277.978}	inf	1321.	6702.65	{1097., 13 803.}	418.814	0.632204	newly	3065.	4226.35	{863., 8417.}	77.6164	0.318665	Reff	1.28034	2.31908	{1.99168, 2.66726}	81.1305	ar	48 598.	90 620.6	{88 395., 92 586.}	86.4698
16	R0	{1.22879, 3.98329}	{1.47651, 5.4396}	{{1.47651, 5.4396}, {1.47651, 5.4396}}	{20.1597, 36.5605}	inf	1186.	4855.15	{3497., 6043.}	309.372	0.598651	newly	2363.	6316.78	{3834., 7454.}	167.32	0.417469	Reff	1.12545	1.05663	{0.844283, 1.28433}	14.2539	ar	34 405.	56 691.1	{55 953., 57 543.}	64.7758
17	R0	{1.34925, 3.64524}	{1.43579, 3.38828}	{{1.43579, 3.38828}, {1.43579, 3.38828}}	{6.41393, 7.04919}	inf	1082.	3843.61	{1482., 5168.}	264.402	0.579809	newly	2774.	8525.35	{4354., 10 696.}	215.506	0.521147	Reff	1.28476	1.15767	{1.00245, 1.355}	12.3837	ar	46 871.	53 839.5	{53 200., 54 612.}	14.8674
18	R0	{1.33054, 2.25275}	{1.51531, 2.72346}	{{1.51531, 2.72346}, {1.51531, 2.72346}}	{13.8868, 20.8949}	inf	387.	1855.26	{619., 4232.}	381.262	0.580463	newly	1979.	7451.48	{2688., 14 437.}	278.637	0.511273	Reff	1.30961	1.42332	{1.29776, 1.48596}	9.60987	ar	44 336.	59 243.	{58 432., 59 864.}	33.6229
19	R0	{1.28394, 3.69859}	{1.47651, 5.4396}	{{1.47651, 5.4396}, {1.47651, 5.4396}}	{14.9984, 47.0723}	inf	1262.	4388.44	{2337., 5902.}	247.737	0.510803	newly	2837.	5978.7	{3798., 7595.}	113.362	0.316911	Reff	1.18844	1.1518	{0.936358, 1.35926}	12.4608	ar	39 558.	56 691.1	{55 953., 57 543.}	43.3114
20	R0	{1.2883, 2.26493}	{1.43965, 2.60601}	{{1.43965, 2.60601}, {1.43965, 2.60601}}	{11.748, 15.0592}	inf	248.	1202.	{136., 2331.}	402.355	0.639086	newly	1268.	4994.96	{798., 9440.}	311.653	0.576958	Reff	1.27318	1.3791	{1.32333, 1.43342}	8.32072	ar	40 768.	54 202.8	{53 240., 55 085.}	32.9544
21	R0	{1.34098, 2.78621}	{1.2663, 1.35135}	{{1.2663, 1.35135}, {1.2663, 1.35135}}	{5.56906, 51.4986}	inf	675.	1409.25	{149., 2399.}	143.574	0.452046	newly	2390.	8344.19	{1232., 12 195.}	263.094	0.536701	Reff	1.30397	1.13318	{0.996844, 1.25219}	13.0974	ar	44 605.	39 254.5	{38 362., 40 803.}	11.9953
22	R0	{1.33783, 5.95911}	{1.38889, 4.25615}	{{1.38889, 4.25615}, {1.38889, 4.25615}}	{3.81663, 28.5774}	inf	2154.	3402.11	{1924., 4302.}	63.8828	0.212224	newly	2912.	5341.18	{2614., 7026.}	87.4792	0.260085	Reff	1.18802	0.961627	{0.771792, 1.1904}	20.1844	ar	45 418.	50 357.8	{49 757., 51 023.}	10.8763
23	R0	{1.33359, 3.3069}	{1.44762, 4.00571}	{{1.44762, 4.00571}, {1.44762, 4.00571}}	{8.5506, 21.1319}	inf	965.	3191.64	{780., 5347.}	242.669	0.502011	newly	2751.	6333.12	{1923., 9075.}	143.536	0.383063	Reff	1.27359	1.24344	{1.0837, 1.41338}	9.14524	ar	46 245.	54 628.9	{53 756., 55 343.}	18.1293
24	R0	{1.30588, 2.56123}	{1.43965, 2.60601}	{{1.43965, 2.60601}, {1.43965, 2.60601}}	{10.2437, 1.74838}	inf	683.	2609.44	{497., 4626.}	297.482	0.561362	newly	2554.	8901.4	{2331., 14 057.}	259.764	0.527699	Reff	1.26499	1.28693	{1.15447, 1.41673}	6.80837	ar	42 383.	54 202.8	{53 240., 55 085.}	27.8882
25	R0	{1.29813, 4.51849}	{1.41571, 4.94504}	{{1.41571, 4.94504}, {1.41571, 4.94504}}	{9.05764, 9.4401}	inf	1351.	3837.48	{2838., 4854.}	190.941	0.455099	newly	2397.	5614.39	{4144., 6885.}	140.875	0.378309	Reff	1.19921	1.09868	{0.914478, 1.26474}	12.5469	ar	43 371.	52 325.9	{51 631., 53 138.}	20.6471
26	R0	{1.33231, 3.99953}	{1.40116, 4.24222}	{{1.40116, 4.24222}, {1.40116, 4.24222}}	{5.16772, 6.06796}	inf	1549.	3507.74	{1152., 4712.}	141.654	0.426544	newly	3228.	5919.74	{2434., 7659.}	97.7294	0.343453	Reff	1.2303	1.10122	{0.922776, 1.34523}	13.9659	ar	44 733.	51 167.2	{50 445., 51 984.}	14.3835
27	R0	{1.30555, 4.61445}	{1.70777, 8.49961}	{{1.70777, 8.49961}, {1.70777, 8.49961}}	{30.8085, 84.1955}	inf	1634.	6500.49	{2564., 9459.}	303.367	0.567954	newly	2878.	5962.15	{2683., 8281.}	117.704	0.335348	Reff	1.20568	1.36677	{1.15841, 1.59694}	15.4225	ar	42 624.	69 566.9	{69 033., 69 995.}	63.2107
28	R0	{1.26819, 5.34969}	{1.38889, 4.25615}	{{1.38889, 4.25615}, {1.38889, 4.25615}}	{9.5175, 20.4412}	inf	1779.	3452.14	{2269., 4340.}	99.2411	0.298837	newly	2612.	5655.82	{3538., 7029.}	120.727	0.339369	Reff	1.1566	1.03949	{0.822098, 1.26281}	14.359	ar	38 946.	50 357.8	{49 757., 51 023.}	29.3016
29	R0	{1.19925, 3.00394}	{1.31023, 3.04996}	{{1.31023, 3.04996}, {1.31023, 3.04996}}	{9.25412, 1.53199}	inf	770.	2364.67	{1410., 3153.}	211.367	0.475095	newly	2181.	5572.48	{4188., 7027.}	159.628	0.406444	Reff	1.12966	1.06924	{0.906967, 1.2237}	10.7085	ar	31 727.	43 460.7	{42 298., 44 573.}	36.9832
30	R0	{1.35749, 3.75483}	{1.56984, 4.94226}	{{1.55618, 4.83753}, {1.59034, 5.09935}}	{15.6431, 31.624}	inf	1440.	3942.49	{185., 7515.}	204.245	0.580173	newly	3590.	6526.97	{344., 10 684.}	114.146	0.459834	Reff	1.28281	1.35647	{1.15331, 1.55119}	10.9405	ar	48 244.	62 796.3	{61 412., 64 214.}	30.164
31	R0	{1.27131, 4.2161}	{1.64839, 7.01474}	{{1.67405, 7.28757}, {1.67405, 7.28757}}	{29.6604, 66.3799}	inf	1285.	5157.88	{1177., 8983.}	312.112	0.593587	newly	2456.	5838.95	{1562., 8934.}	155.235	0.432748	Reff	1.18819	1.35273	{1.04531, 1.61738}	18.7467	ar	38 299.	65 849.8	{51 157., 68 480.}	71.9361
32	R0	{1.31827, 6.18119}	{1.38889, 4.25615}	{{1.38889, 4.25615}, {1.38889, 4.25615}}	{5.35702, 31.1435}	inf	2473.	3402.11	{1924., 4302.}	46.9802	0.171876	newly	3092.	5341.18	{2614., 7026.}	78.2284	0.241479	Reff	1.16063	0.961627	{0.771792, 1.1904}	18.8699	ar	45 262.	50 357.8	{49 757., 51 023.}	11.2584
33	R0	{1.20398, 1.35167}	{1.40724, 2.57945}	{{1.4031, 2.55684}, {1.4031, 2.55684}}	{16.882, 90.834}	inf	104.	811.269	{40., 1895.}	698.78	0.827366	newly	1068.	3486.38	{228., 7914.}	254.646	0.578568	Reff	1.19578	1.36715	{1.31424, 1.40094}	14.3311	ar	33 329.	51 773.8	{50 618., 52 281.}	55.3417
34	R0	{1.32776, 3.146}	{1.74706, 5.59272}	{{1.74592, 5.58477}, {1.74592, 5.58477}}	{31.5798, 77.7723}	inf	1228.	6172.78	{3212., 9497.}	404.196	0.666964	newly	3687.	9513.85	{5888., 13 026.}	160.599	0.401793	Reff	1.25318	1.44358	{1.20013, 1.59081}	16.7774	ar	44 872.	71 059.3	{70 636., 71 495.}	58.36
35	R0	{1.279, 1.11336}	{1.27121, 1.91565}	{{1.27121, 1.91565}, {1.27121, 1.91565}}	{0.60907, 72.0603}	inf	39.	404.826	{43., 1203.}	953.623	0.916243	newly	625.	2207.52	{207., 5968.}	285.273	0.693009	Reff	1.27363	1.24588	{1.19672, 1.26845}	2.17852	ar	41 033.	37 950.	{38 243., 41 018.}	7.77593
36	R0	{1.20029, 1.94717}	{1.51531, 2.72346}	{{1.51531, 2.72346}, {1.51531, 2.72346}}	{26.2453, 39.8676}	inf	272.	1855.26	{619., 4232.}	582.08	0.724615	newly	1470.	7451.48	{2688., 14 437.}	406.904	0.630335	Reff	1.18231	1.42332	{1.29776, 1.48596}	20.3851	ar	33 234.	59 243.	{58 432., 59 864.}	78.2603
37	R0	{1.33216, 3.69685}	{1.54465, 5.30296}	{{1.54465, 5.30296}, {1.54465, 5.30296}}	{15.9508, 43.4454}	inf	1487.	4556.5	{1501., 7240.}	210.28	0.438165	newly	3432.	6569.32	{2813., 8989.}	100.908	0.288285	Reff	1.23359	1.24821	{0.930991, 1.4731}	13.5843	ar	45 235.	61 243.5	{60 362., 61 965.}	35.3896
38	R0	{1.25434, 2.04314}	{1.43965, 2.60601}	{{1.43965, 2.60601}, {1.43965, 2.60601}}	{14.7735, 27.5493}	inf	320.	1202.	{136., 2331.}	299.725	0.579192	newly	1505.	4994.96	{798., 9440.}	254.158	0.537102	Reff	1.23694	1.3791	{1.32333, 1.43342}	11.4927	ar	38 662.	54 202.8	{53 240., 55 085.}	40.1967
39	R0	{1.27466, 3.0741}	{1.4804, 3.12918}	{{1.4804, 3.12918}, {1.4804, 3.12918}}	{16.1408, 1.79174}	inf	1446.	3782.85	{588., 5895.}	182.392	0.470878	newly	3912.	9398.85	{2400., 13 150.}	156.824	0.428852	Reff	1.18848	1.21043	{0.929114, 1.44962}	13.7595	ar	40 181.	57 005.2	{56 101., 57 732.}	41.8711
40	R0	{1.33045, 2.68154}	{1.43965, 2.60601}	{{1.43965, 2.60601}, {1.43965, 2.60601}}	{8.20775, 2.81667}	inf	888.	2609.44	{497., 4626.}	215.36	0.494402	newly	3300.	8901.4	{2331., 14 057.}	185.63	0.451803	Reff	1.28496	1.28693	{1.15447, 1.41673}	6.6884	ar	45 951.	54 202.8	{53 240., 55 085.}	17.9579
41	R0	{1.37003, 2.83984}	{1.4023, 2.07266}	{{1.4023, 2.07266}, {1.4023, 2.07266}}	{2.35542, 27.0149}	inf	521.	1835.48	{359., 3998.}	264.612	0.510578	newly	2106.	8954.63	{2254., 15 217.}	331.489	0.585398	Reff	1.34277	1.29718	{1.15691, 1.38369}	4.74818	ar	48 926.	51 336.4	{50 349., 52 433.}	4.92671
42	R0	{1.3003, 2.94885}	{1.6079, 4.3716}	{{1.53501, 4.02511}, {1.61691, 4.41442}}	{23.6562, 48.2475}	inf	940.	3941.13	{306., 7269.}	337.545	0.610251	newly	2995.	7823.08	{757., 12 868.}	183.291	0.474703	Reff	1.24816	1.40161	{1.20617, 1.60404}	14.6251	ar	44 237.	64 605.8	{60 572., 65 743.}	46.0447
43	R0	{1.33756, 2.92653}	{1.50627, 2.6714}	{{1.51531, 2.72346}, {1.51531, 2.72346}}	{12.6132, 8.71797}	inf	626.	1888.24	{629., 4232.}	205.731	0.410186	newly	1978.	7770.03	{3149., 14 437.}	293.567	0.536652	Reff	1.31066	1.41019	{1.26293, 1.48511}	8.70386	ar	47 342.	58 494.9	{58 024., 59 857.}	23.5581
44	R0	{1.35801, 2.92276}	{1.54098, 3.31039}	{{1.54098, 3.31039}, {1.54098, 3.31039}}	{13.4734, 13.2625}	inf	645.	2157.9	{502., 4280.}	251.323	0.491544	newly	2455.	6633.35	{1932., 11 952.}	187.019	0.432522	Reff	1.32668	1.43707	{1.32447, 1.52019}	8.99455	ar	47 940.	60 965.5	{60 473., 61 629.}	27.1704
45	R0	{1.31342, 4.02649}	{1.43206, 3.35359}	{{1.43579, 3.38828}, {1.43579, 3.38828}}	{9.03294, 16.7118}	inf	1370.	4203.16	{2683., 4972.}	214.082	0.517872	newly	2863.	8378.12	{6723., 10 364.}	199.628	0.495154	Reff	1.22662	1.01428	{0.860332, 1.27524}	19.5887	ar	42 995.	53 530.5	{52 666., 54 612.}	24.504
46	R0	{1.37919, 3.75889}	{1.47488, 4.06661}	{{1.47488, 4.06661}, {1.47488, 4.06661}}	{6.93813, 8.18646}	inf	1025.	3273.19	{885., 5065.}	232.889	0.521778	newly	2667.	6677.88	{2239., 9535.}	164.104	0.436947	Reff	1.32725	1.28587	{1.16079, 1.43115}	7.04925	ar	49 157.	56 717.4	{55 905., 57 514.}	15.3801
47	R0	{1.37458, 3.56117}	{1.47608, 3.83841}	{{1.47608, 3.83841}, {1.47608, 3.83841}}	{7.38407, 7.78508}	inf	1179.	4182.5	{1091., 6164.}	260.217	0.518625	newly	3193.	8521.12	{2928., 10 930.}	172.031	0.414825	Reff	1.31164	1.20911	{1.00375, 1.42116}	10.8164	ar	48 478.	56 853.7	{56 175., 57 621.}	17.2774
48	R0	{1.33393, 3.78296}	{1.87503, 10.2266}	{{1.87584, 10.2217}, {1.88056, 10.2502}}	{40.5647, 170.334}	inf	1368.	5535.87	{1013., 10 979.}	314.959	0.552546	newly	3101.	4465.02	{940., 8071.}	77.041	0.296765	Reff	1.2473	1.6166	{1.31331, 1.83369}	29.6076	ar	44 885.	75 840.4	{75 332., 76 312.}	68.966
49	R0	{1.24918, 2.39046}	{1.27121, 1.91565}	{{1.27121, 1.91565}, {1.27121, 1.91565}}	{1.76356, 19.8627}	inf	662.	1781.09	{725., 2427.}	176.408	0.432844	newly	2523.	6732.82	{3829., 8943.}	173.124	0.430245	Reff	1.19869	1.06943	{0.890826, 1.21978}	11.8029	ar	35 969.	39 671.	{38 472., 41 018.}	10.2922
50	R0	{1.28281, 2.3032}	{1.4804, 3.12918}	{{1.4804, 3.12918}, {1.4804, 3.12918}}	{15.4029, 35.8623}	inf	746.	2526.39	{214., 5764.}	263.364	0.550749	newly	2869.	7113.45	{818., 12 926.}	172.727	0.465254	Reff	1.23601	1.33434	{1.13868, 1.46727}	11.734	ar	40 223.	57 005.2	{56 101., 57 732.}	41.7229
